# Elevated platelet-to-lymphocyte ratio is associated with increased breast cancer risk: A cross-sectional analysis of the NHANES 2009 to 2020

**DOI:** 10.1097/MD.0000000000049311

**Published:** 2026-06-19

**Authors:** Yanting Li, Yanpeng Li, Xurui Li, Peiling Zhu, Mengsi Zhou

**Affiliations:** a Department of Breast and Thyroid Surgery, The Second Hospital of Hebei Medical University, Shijiazhuang, Hebei, P.R. China; b Department of Emergency, Hebei General Hospital, Shijiazhuang, Hebei, P.R. China.

**Keywords:** breast cancer, NHANES, platelet-to-lymphocyte ratio, Systemic Immune Inflammation Index, systemic immune inflammatory biomarkers

## Abstract

Inflammation is a recognized driver of tumorigenesis, particularly in breast cancer. The platelet-to-lymphocyte ratio (PLR) has emerged as a readily available inflammatory indicator; however, its independent association with breast cancer risk in a nationally representative civilian cohort has not been quantified. We aimed to evaluate the cross-sectional relationship between PLR and breast cancer prevalence among U.S. adults and to examine whether this association is modified by sociodemographic or clinical factors. We analyzed 14,152 women aged ≥20 years from the continuous National Health and Nutrition Examination Survey cycles from to 2009 to 2020. Breast cancer cases (n = 419) were defined using a self-reported physician diagnosis. We excluded male participants and individuals with missing data on blood cell counts (platelets, neutrophils, lymphocytes) or key covariates. After natural-log transformation, PLR and systemic immune-inflammation index (SII) were entered as primary exposures. Multivariable logistic regression was adjusted for age, race/ethnicity, body mass index, smoking, alcohol, age at menarche, parity, hormone use history, poverty-income ratio, and comorbidities. Generalized additive models with penalized splines and 2-piece logistic regression were fitted to detect threshold effects. Subgroup, interaction, and leave-one-out sensitivity analyses were performed to test the robustness. Each 1-unit increase in ln-PLR was independently associated with 78 % higher odds of breast cancer (OR = 1.78; 95 % CI 1.00–3.17; *P* = .050), whereas ln-SII showed no significant association (OR = 1.24; 95 % CI 0.87–1.78; *P* = .221). A significant interaction was observed between the ln-PLR and the family monthly poverty level category (Pinteraction < 0.05); no effect modification was detected for age, race, obesity metrics, socioeconomic status, reproductive variables, or comorbidities (all Pinteraction > 0.05). Threshold analysis revealed no evidence of a nonlinear inflection point. Elevated PLR was independently associated with increased breast cancer prevalence in a large nationally representative sample, while SII did not show a significant association. This supports PLR’s potential as an inexpensive inflammatory biomarker for risk stratification.

## 1. Introduction

Breast cancer holds the distinction of being the most prevalent malignant tumor among females globally. Although lung cancer surpasses breast cancer incidence among women by 2023, breast cancer continues to rank second in mortality among female malignancies, trailing only lung cancer.^[1]^ Chronic inflammation has been identified as a pivotal factor driving both the initiation and advancement of cancer,^[2]^ potentially facilitating early tumorigenesis through the action of pro-inflammatory cytokines and reactive oxygen species. In breast cancer, inflammation has been implicated in activating the NF-κB signaling pathway, which may promote the emergence of more aggressive tumor subtypes.^[3]^

The hallmarks of cancer include tumor-promoting inflammation and immune system function in monitoring and eliminating malignant cells, which supports the survival and multiplication of these cells.^[4,5]^ Systemic inflammatory responses affect cancer development and progression at the molecular level and involve mechanisms such as DNA damage and cellular proliferation.^[6]^ Furthermore, in addition to tumor cells, immune and inflammatory cells such as neutrophils, monocytes, platelets, and lymphocytes play a role in the infiltration of malignant cells into peripheral blood, aiding their survival and establishment in distant organs.^[7]^ Various inflammation- and immunity-based indicators, such as lymphocyte count, neutrophil-to-lymphocyte ratio, monocyte-to-lymphocyte ratio, and platelet-to-lymphocyte ratio (PLR), have been employed to predict survival outcomes in cancer patients.^[8–11]^ The systemic immune-inflammation index (SII), calculated using lymphocyte, neutrophil, and platelet counts, reflects the balance between host inflammatory and immune status and serves as a well-established prognostic factor in multiple malignancies.^[12–16]^

However, the predictive value of the SII and PLR in assessing the risk of breast cancer remains unclear. The primary aim of this study was to perform an exhaustive examination of the relationship between the Systemic Immune-Inflammation Index (SII) and the PLR with respect to their potential to predict the risk of developing breast cancer. We conducted a thorough and robust analysis employing a cross-sectional study design and examining a substantial representative sample drawn from the national population. The central focus of this study was to elucidate the potential of these markers as diagnostic indicators of breast cancer, thereby contributing to a better understanding of their role in the disease.

## 2. Methods

### 2.1. Study design and population

The National Health and Nutrition Examination Survey (NHANES), an ongoing cross-sectional study in the United States, is a vital resource for the systematic collection of health-related data at the national level. The National Center for Health Statistics (NCHS) Ethics Review Board approved the NHANES protocol, and all the study procedures adhered to the principles outlined in the Declaration of Helsinki. Written informed consent was obtained from all participants prior to data collection (https://www.cdc.gov/nchs/nhanes/about/erb.html?CDC_AAref_Val). The sample size of the NHANES survey depended on the number of participants enrolled in the study. NHANES surveys span a diverse range of crucial domains, including demographics, socioeconomic indicators, dietary behaviors, and health-related insights. The official NHANES website (https://www.cdc.gov/nchs/index.htm) provides a comprehensive outline of the multilevel and intricate sampling techniques employed in the data-collection process. Our study recruited 59,842 participants from 5 cycles of the NHANES, spanning the years 2009 to 2010, 2011 to 2012, 2013 to 2014, 2015 to 2016, and 2017 to 2020. We excluded the following participants: males; individuals under 20 years of age; those with missing data on complete blood count (CBC) parameters (platelets, neutrophils, lymphocytes) necessary to calculate SII and PLR; those with missing data on breast cancer status or key covariates (e.g., BMI, smoking status). After applying these exclusions, a final subset of 14,152 women aged ≥20 years was included in the analysis.

### 2.2. Ethical review

The NCHS Ethics Review Board approved the NHANES protocol (Continuation of Protocol #2011–17, #2018-01). All study procedures adhered to the principles outlined in the Declaration of Helsinki. Written informed consent was obtained from all participants prior to data collection.

### 2.3. Outcomes

The primary focus of our study was the diagnosis of gynecological cancer. Cancer types were identified through responses to specific questions on the Medical Status Questionnaire: “Has a doctor or health professional ever informed you that you have cancer or malignancy?” and “What type of cancer was it specific?” Participants diagnosed with breast cancer alone were categorized separately as outcome variables.

### 2.4. Systemic immune-inflammatory biomarkers (SII and PLR)

Fasting blood samples were collected from participants during the MEC visit by trained phlebotomists following a standardized venipuncture protocol. A CBC with differential, including platelet, neutrophil, and lymphocyte counts, was performed on automated hematology analyzers at the NHANES contract laboratories, which adhere to the quality assurance and control protocols mandated by the Centers for Disease Control and Prevention (CDC) and are certified under the CLIA. Detailed specimen collection, processing, and analytical procedures are documented in the NHANES Laboratory/Medical Technologists Procedures Manual. The systemic immune-inflammation index (SII) and PLR were calculated using the following formulas: SII = (platelet count × neutrophil count)/ lymphocyte count and PLR = platelet count / lymphocyte count, where all cell counts were derived from the CBC differential as absolute values (cells/μL).

### 2.5. Covariates

Drawing upon both existing literature and clinical expertise, we incorporated a range of covariates in our study: age, race (categorized within the NHANES framework as Mexican American, Other Hispanic, Non-Hispanic White, Non-Hispanic Black, and Other Race), educational attainment (less than high school, high school or equivalent, and some college or more), body mass index (BMI; categorized as < 25, 25.0–29.9, and ≥30.0 kg/m^2^), waist circumference, family monthly poverty level (categorized as low ≤ 1.3, medium 1.3–1.85, and high > 3.5), age at menarche, use of estrogen-containing medications, childbearing history, hypertension (defined by self-report, prescription medication use, mean diastolic blood pressure ≥80 mm Hg and/or mean systolic blood pressure ≥130 mm Hg), hyperlipidemia (defined as serum total cholesterol ≥5.72 mmol/L, triglycerides ≥1.70 mmol/L, or low-density lipoprotein ≥4.1 mmol/L), diabetes (determined by self-report, insulin or diabetes medication use, glycated hemoglobin or hemoglobin A1c > 6.5%, and/or fasting plasma glucose ≥7.0 mmol/L), and the Systemic Immune-Inflammation Index (SII) and PLR. Furthermore, we used the Patient Health Questionnaire-9 (PHQ-9) to assess and quantify depressive symptoms in patients with cancer. The PHQ-9 consists of 9 items, each rated on a scale of 0 to 3, resulting in a composite score ranging from 0 to 27. Higher scores are indicative of increased severity of depressive symptoms, allowing for the classification of patients into 3 distinct categories: those without depression (scoring 0–4), those with mild depression (scoring 5–9), and those with moderate-to-severe depression (score ≥10).^[17]^

### 2.6. Statistical analyses

Continuous variables are represented as mean values with SD, whereas categorical variables are expressed as frequencies and corresponding percentages. For comparative assessments of baseline characteristics across groups, weighted t-tests were used for continuous variables, and weighted chi-square tests were applied to categorical variables. Given the skewed distributions of the Systemic Immune-Inflammation Index (SII) and PLR, a logarithmic transformation using the natural logarithm (ln) was implemented to approximate normal distributions. The transformed variables were stratified into quartiles (Q1, Q2, Q3, and Q4). Initially, a multifactorial logistic regression analysis was conducted to investigate the impact of the SII and PLR on breast cancer risk. Additionally, for the sensitivity analysis, the ln-transformed SII and PLR were treated as categorical variables (quartiles), with the lowest quartile (Q1) serving as the reference group. The analysis was repeated using multifactorial logistic regression, and the results were presented as odds ratios (ORs) with 95% confidence intervals (CIs). For comparisons among the 4 groups regarding SII and PLR, Bonferroni correction was applied (adjusted α level = 0.05/3 ≈ 0.0167), and p-values for trend tests were reported separately without inclusion in the correction. A trend test was also conducted to assess the linear relationships. In our study, 3 models were constructed to account for the various confounding factors. The initial model remained unaltered, whereas model 1 incorporated adjustments for age and race. Model 2 was further refined by adjusting for all covariates as outlined in Model 1. Additionally, to investigate potential nonlinear relationships between the Systemic Immune-Inflammation Index (SII) and PLR with respect to breast cancer risk, we used restricted cubic spline (RCS) regression analysis. The likelihood ratio test was used to detect nonlinearity. In cases where nonlinearity was identified, a 2-stage segmented regression analysis was conducted using inflection point values to investigate the threshold effects of the independent variables on breast cancer risk. Interaction and subgroup analyses were conducted to assess whether these relationships were influenced by age, race, education, BMI, waist circumference, monthly family poverty level category, hypertension, hyperlipidemia, diabetes, and PHQ-9 score. In these analyses, the SII and PLR were considered continuous and categorical variables (quartiles). For subgroup analyses and interaction tests, we applied false discovery rate correction using the Benjamini-Hochberg method, controlling false discovery rate at ≤.05. Interactions with adjusted *P*-values <.05 were considered statistically significant. To address the complex survey design characteristics, we applied sample weights to adjust for unequal selection probabilities and nonresponse bias in all analyses, in strict accordance with the NHANES weighting methodology guidelines (https://wwwn.cdc.gov/nchs/nhanes/analyticguidelines.aspx#estimation-and-weighting-procedures).. All statistical analyses were performed using R software (https://www.r-project.org/; version 4.2.1). Statistical significance was defined as a 2-tailed *P*-value < 0.05.

## 3. Results

### 3.1. Characteristics of included participants

**Figure 1.**
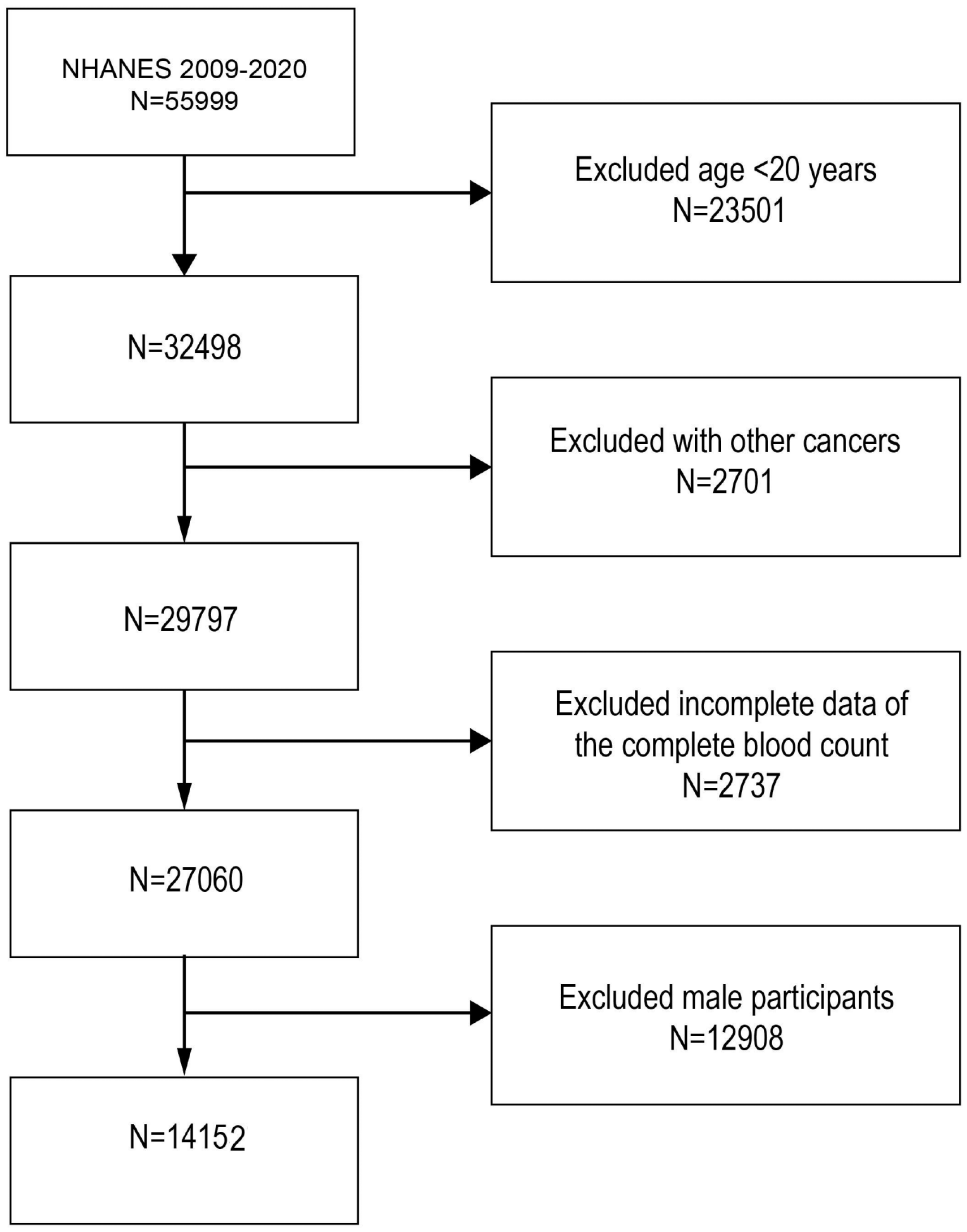
Flowchart of the sample selection from NHANES 2009 to 2020. NHANES = National Health and Nutrition Examination Survey.

**Figure 2.**
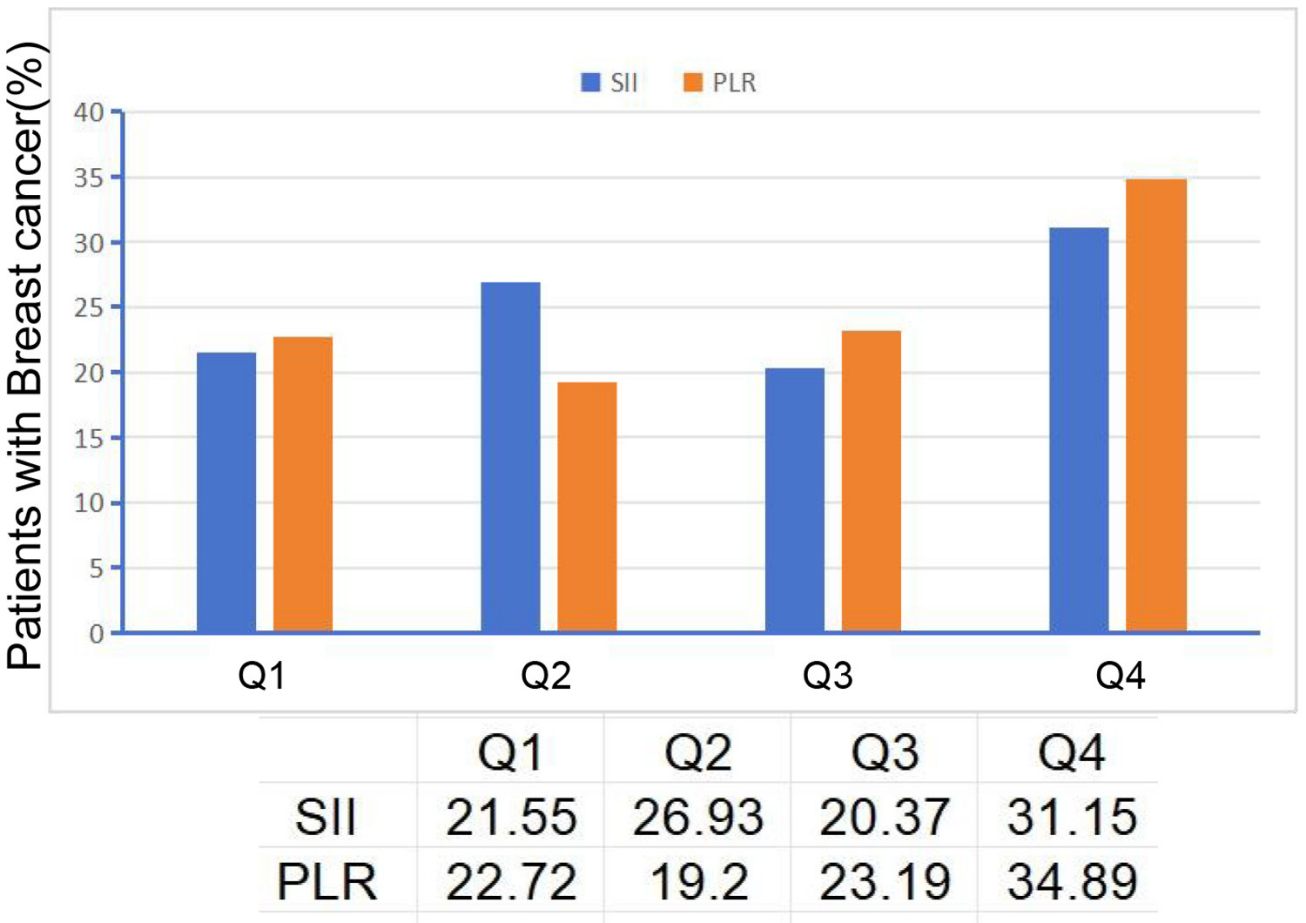
The proportion of patients with breast cancer sorted by quartiles of ln-transformed SII and PLR. PLR = platelet-to-lymphocyte ratio, SII = Systemic Immune-Inflammation Index.

The participant selection process is detailed in Figure 1, resulting in a final analytical sample of 14,152 women. The baseline characteristics of the cohort are summarized in Table 1. Overall, the mean age was 47.8 years. The majority of participants were Non-Hispanic White (63.39%). Compared to participants without breast cancer (n = 13,725), those diagnosed with breast cancer (n = 427) were significantly more likely to have obesity (BMI ≥30 kg/m^2^: 47.94% vs 39.60%, *P* = .004) and had a larger mean waist circumference (101.83 cm vs 97.21 cm, *P* < .001). The prevalence of hypertension (43.91% vs 35.35%, *P* = .034) and diabetes (15.97% vs 11.64%, *P* = .020) was also significantly higher in the breast cancer group. Furthermore, breast cancer patients had a significantly higher mean PLR (139.62 vs 125.33, *P* = .017). No statistically significant differences were observed between the 2 groups in terms of age distribution, race/ethnicity, education level, family poverty level, hyperlipidemia, depressive symptoms (PHQ-9 score), reproductive history, age at menarche, or use of estrogen medications (all *P* > .05). The difference in the Systemic Immune-Inflammation Index (SII) was not statistically significant (*P* = .119). Furthermore, Figure 2 illustrates the distribution of breast cancer patients according to the quartiles of the natural logarithm (ln)-transformed Systemic Immune-Inflammation Index (SII) and PLR. Notably, a higher prevalence of breast cancer was observed in individuals belonging to the higher quartiles of both SII and PLR.

**Table 1:** Characteristics of the study population.

Characteristics	Overall (n = 14,152)	Non BCa (n = 13,725)	BCa (n = 427)	*P*-value
Age, years, n(%)				
20–39	5249 (37.09%)	5103 (37.18%)	146 (34.31%)	.387
40–59	5140 (36.32%)	4989 (36.35%)	151 (35.32%)	
≥60	3763 (26.59%)	3633 (26.46%)	130 (30.37%)	
Race, n(%)				
Mexican American	1205 (8.52%)	1175 (8.56%)	30 (7.02%)	.788
Other Hispanic	959 (6.78%)	928 (6.76%)	31 (7.35%)	
Non-Hispanic White	8971 (63.39%)	8692 (63.33%)	279 (65.35%)	
Non-Hispanic Black	1753 (12.39%)	1702 (12.40%)	51 (11.89%)	
Other Races	1264 (8.93%)	1228 (8.94%)	36 (8.39%)	
Education level, n(%)				
Less than high school	721 (5.09%)	701 (5.11%)	20 (4.63%)	.426
High school or equivalent	4413 (31.18%)	4266 (31.08%)	147 (34.40%)	
Some college or more	9018 (63.72%)	8758 (63.81%)	260 (60.97%)	
BMI, n(%)				
<25 kg/m^2^	4510 (31.87%)	4413 (32.15%)	97 (22.83%)	.004
25–30 kg/m^2^	4002 (28.28%)	3877 (28.25%)	125 (29.23%)	
≥30 kg/m^2^	5640 (39.85%)	5435 (39.60%)	205 (47.94%)	
WC (cm)	98.10 ± 17.05	97.21 ± 16.52	101.83 ± 19.49	<.001
FMPLC, n(%)				
≤1.30	3820 (26.99%)	3685 (26.85%)	135 (31.72%)	.242
1.30–1.85	1795 (12.68%)	1746 (12.72%)	49 (11.59%)	
>1.85	8536 (60.32%)	8294 (60.43%)	242 (56.69%)	
Hypertension, n (%)				
Yes	5039 (35.61%)	4852 (35.35%)	187 (43.91%)	.034
No	9113 (64.39%)	8873 (64.65%)	240 (56.09%)	
Hyperlipidemia, n (%)				
Yes	6616 (46.75%)	6396 (46.60%)	220 (51.45%)	.070
No	7536 (53.25%)	7329 (53.40%)	207 (48.55%)	
Diabetes, n (%)				
Yes	1666 (11.77%)	1598 (11.64%)	68 (15.97%)	.020
No	12,486 (88.23%)	12,127 (88.36%)	359 (84.03%)	
PHQ-9 score, n(%)				
0–4	9308 (65.77%)	9045 (65.90%)	263 (61.61%)	.419
5–9	2259 (15.96%)	2180 (15.88%)	79 (18.44%)	
≥10	2585 (18.27%)	2500 (18.22%)	85 (19.95%)	
Childbearing history				
Yes	13,290(93.91%)	12,903(94.01%)	387 (90.64%)	.166
No	862 (6.09%)	822 (5.99%)	40 (9.36%)	
Age of menarche	12.71 ± 1.80	12.70 ± 1.84	12.65 ± 1.61	.564
Use of estrogen medications				
Yes	2652 (18.74%)	2568 (18.71%)	84 (19.63%)	.761
No	11,500(81.26%)	11,157(81.29%)	343 (80.37%)	
SII	536.01 ± 333.63	536.71 ± 325.86	617.19 ± 434.29	.119
PLR	125.26 ± 48.86	125.33 ± 49.85	139.62 ± 60.34	.017

BCa = breast cancer, BMI = body mass index, FMPLC = family monthly poverty level category, PHQ-9 = Patient Health Questionnaire-9, PLR = platelet-to-lymphocyte ratio, SII = Systemic Immune-Inflammation Index, WC = Waist circumference.

### 3.2. Association of SII and PLR with breast cancer risk

Table 2 and Table S1, Supplemental Digital Content 1 present the associations between the Systemic Immune-Inflammation Index (SII) and PLR with the risk of breast cancer. To rigorously evaluate these relationships, we developed 3 models, each incorporating various confounding variables. Upon comprehensive adjustment for all confounders in Model 2, a statistically significant positive correlation was observed between the ln-transformed PLR and breast cancer risk, with an OR of 1.78 (95% CI: 1.00–3.17, *P* = .050). Conversely, in the final model, the relationship between ln (SII) and breast cancer risk failed to reach statistical significance, yielding an OR of 1.24 (95% CI: 0.87–1.78, *P* = .221). Consistent with these findings, a similar trend was evident when the ln-transformed SII and PLR were treated as categorical variables (quartiles) in sensitivity analysis. Specifically, within the fully adjusted model (Model 2), a gradual increase in breast cancer risk was noted across the quartiles of PLR, with the highest quartile (Q4) exhibiting a significantly elevated risk compared to the lowest quartile (Q1) (*P* for trend <.05).

**Table 2:** The relationship between ln-transformed PLR and the risk of breast cancer.

Characteristic	Crude model*		Model 1†		Model 1‡	
	OR (95% CI)	*P*-value	OR (95% CI)	*P*-value	OR (95% CI)	*P*-value
PLR (ln-transformed)	1.73 (1.09–2.78)	.023	1.67 (1.01–2.74)	.046	1.78 (1.00–3.17)	.050
In(PLR) (quartile)						
Q1	Ref		Ref		Ref	
Q2	0.73 (0.49–1.08)	.119	0.74 (0.49–1.13)	.178	0.71 (0.41–1.26)	.251
Q3	1.02 (0.70–1.51)	.888	1.04 (0.69–1.55)	.862	1.07 (0.57–2.05)	.821
Q4	1.59 (1.13–2.23)	.009	1.57 (1.08–2.27)	.018	1.80 (1.04–3.14)	.043
*P* for trend	–	.002	–	.006	–	.022

CI = confidence interval, OR = odds ratio, PLR = platelet-to-lymphocyte ratio, Q = quartile, SII = Systemic Immune-Inflammation Index.

* The crude model was not adjusted for any covariates.

† Model 1 was adjusted for age and race.

‡ Model 2 was adjusted for all covariates based on model 1.

### 3.3. Smooth curve fitting for SII and PLR with breast cancer risk

**Figure 3.**
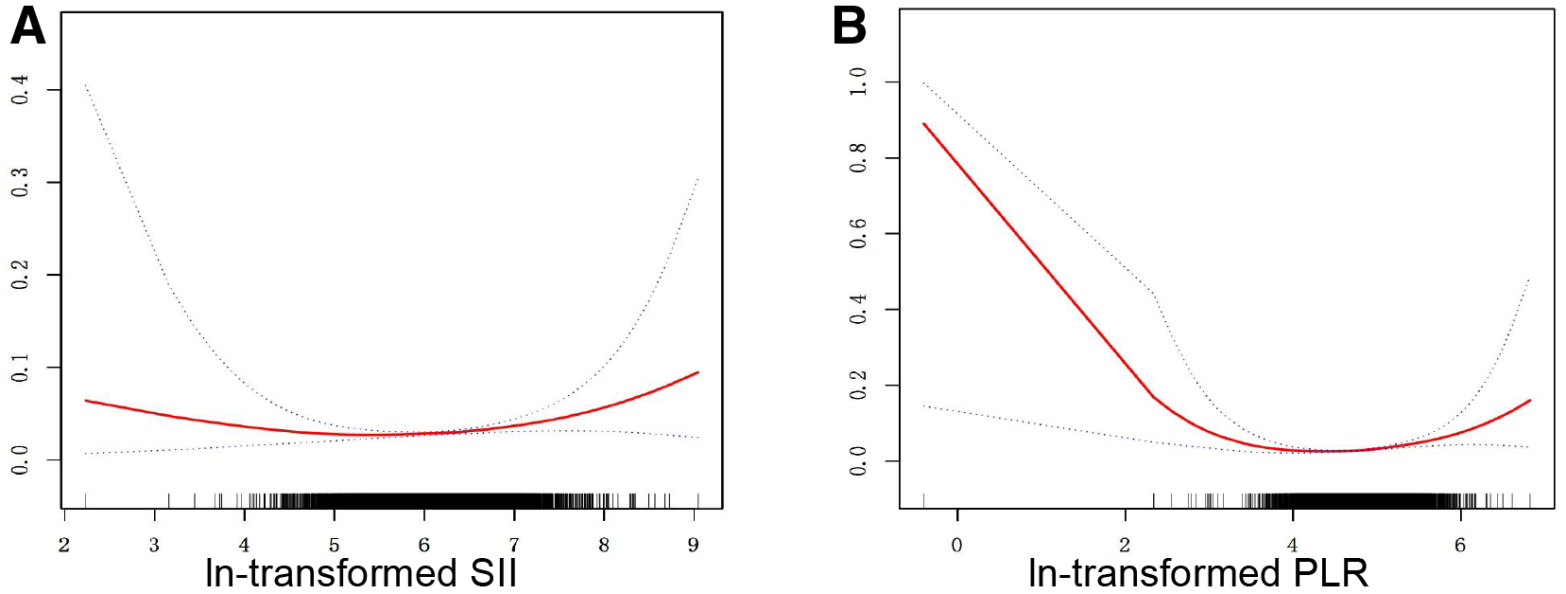
Smooth curve fitting for SII and PLR with breast cancer. (A) SII and breast cancer; (B) PLR and breast cancer. PLR = platelet-to-lymphocyte ratio, SII = Systemic Immune-Inflammation Index.

To further validate the robustness of our findings, we explored the potential for a nonlinear relationship among the Systemic Immune-Inflammation Index (SII), PLR, and breast cancer risk. As illustrated in Figure 3, using a RCS regression model that accounted for all confounding factors, a robust nonlinear correlation was observed between ln (PLR) and breast cancer risk using a RCS regression model that accounted for all confounding factors risk. Intriguingly, employing smoothing curve fitting with a penalized spline approach, we uncovered an “L”-shaped association between ln (PLR) and breast cancer. Subsequently, we conducted segmented regression and threshold analyses, as presented in Table 3, which revealed an inflection point for ln (PLR) at 4.32. Notably, when ln (PLR) was below this threshold, each incremental unit was associated with a 77% decrease in the risk of breast cancer (OR = 0.23, 95% CI: 0.09–0.62, *P* = .003). Conversely, when ln (PLR) surpassed 4.32, each additional unit led to a 134% increase in breast cancer risk (OR = 2.34, 95% CI: 1.57–3.49, *P* <.001). These findings underscore the complex and non-monotonic nature of the relationship between ln (PLR) and breast cancer risk.

**Table 3:** The threshold effect of ln-transformed PLR on breast cancer was analyzed using a 2-stage phased regression model.

Models	Adjusted OR (95% CI), *P*-value
Model I	
Logistic regression (the standard linear model)	1.74 (1.19–2.53) .004
Model II	
Inflection point	4.32
<4.32	0.23 (0.09–0.62) .003
>4.32	2.34 (1.57–3.49) <.001
*P* for Log-likelihood ratio	.001

### 3.4. Subgroup analyses and sensitivity analyses

Subgroup analyses were conducted for various factors, including age, race, education level, waist circumference, family monthly poverty level category, use of estrogen-containing medications, childbearing history, hypertension, hyperlipidemia, diabetes, and the PHQ-9 score (Table S2, Supplemental Digital Content 2). The results demonstrated a significant interaction between the ln-transformed PLR and the family monthly poverty level category (*P* for interaction = .031), whereas no significant interactions were observed in other subgroup analyses (*P* for interaction >.05).

## 4. Discussion

The peripheral blood inflammatory index (SII) was initially devised to reflect the extent of systemic inflammation and stress in critically ill patients. However, it has subsequently been proven to possess predictive and prognostic values across a broad spectrum of diseases,^[18,19]^ marked by its ease of accessibility, cost-effectiveness, reproducibility, and noninvasiveness. Currently, research investigating the predictive potential of SII and PLR for breast cancer risk is scarce, and the findings remain inconclusive. Rajakumar et al reported lower PLR expression in breast cancer cases than in non-breast cancer cases.^[20]^ Conversely, Zha et al failed to discern significant differences in PLR and SII levels between breast cancer and non-breast cancer patients.^[21]^ Furthermore, Aida Alizamir findings contradicted Rajakumar observations, revealing elevated PLR expression in breast cancer compared to non-breast cancer scenarios.^[22]^ our knowledge, this is the first study to examine the relationship between SII and PLR in breast cancer in a broader American population, thereby emphasizing the robustness and reproducibility of our findings.

Our study revealed that the Systemic Immune-Inflammation Index (SII) and PLR in the peripheral blood of adult female breast cancer patients in the United States were significantly higher than those in patients without breast cancer. To facilitate statistical analysis, we further normalized the SII and PLR using natural logarithm (ln) transformation. The results demonstrated a statistically significant positive correlation between the PLR and breast cancer, emphasizing that elevated PLR values are associated with an increased risk of breast cancer. However, the correlation between the Systemic Immune-Inflammation Index (SII) and breast cancer was not statistically significant (*P* >.05). Categorical analysis of log-transformed SII values revealed important findings. While the general trend across quartiles showed consistency, we identified a statistically meaningful disparity when comparing extreme quartiles (Q4 vs Q1). These observations provide additional evidence for the relationship between systemic inflammatory markers and breast cancer susceptibility. The SII is a composite measure that captures the complex interactions between neutrophils, lymphocytes, and platelets during systemic inflammatory processes. Although its clinical utility may be limited by certain biological considerations, this tripartite cellular index offers insights into overall immune homeostasis. Notably, the inclusion of neutrophils, which are highly responsive to acute physiological stressors, such as infection or tissue injury, may reduce the specificity of the index relative to simpler biomarkers, such as PLR. Consequently, the PLR may offer greater specificity as an inflammatory indicator, as reported in previous studies. Our findings suggest that among adult female populations in the United States, PLR is a better predictor of breast cancer than SII. In adult American women with a higher PLR, the potential risk of breast cancer should not be overlooked. Our study has several limitations. First, as a cross-sectional analysis, causal inference cannot be established. Second, breast cancer diagnosis and some covariates were based on self-report, which may be subject to recall bias. Third, the NHANES database lacks information on breast cancer molecular subtyping and detailed disease progression (clinical or pathologic staging), which are important factors in breast cancer research and could potentially influence the relationship with inflammatory markers. Fourth, we only had a single measurement of SII and PLR, precluding the assessment of their dynamic changes over time or their longitudinal impact on breast cancer risk. Fifth, due to the lack of long-term follow-up, we could not determine the relationship between PLR and breast cancer prognosis. These critical questions warrant further investigation through prospective cohort studies using serial biomarker measurements. Furthermore, because this was a cross-sectional study, our design precludes causal inferences. While an elevated PLR is significantly associated with breast cancer risk, we cannot determine whether this elevation precedes tumor development or is a consequence of tumor-induced inflammation. To delineate the exact temporal sequence and establish potential causal mechanisms, well-designed prospective cohort studies incorporating serial PLR measurements during the prediagnostic period are warranted.

Platelets play multifaceted roles in cancer progression. They can deliver various angiogenic factors to tumors and stimulate tumor cells to express angiogenic factors.^[23]^ Furthermore, they form a protective barrier around tumor cells, shielding them from immune attack by lymphocytes.^[24]^ Platelets also protect cancer cells from shear stress and natural killer cells, thereby facilitating cancer metastasis and progression.^[25]^ By releasing inflammatory mediators that support cancer cell survival and proliferation, platelets contribute to a pro-inflammatory microenvironment within tumors.^[25]^ Lymphocytes, the smallest white blood cells, play a pivotal role in innate immune defense against malignancies. A relative decrease in lymphocytes indicates a diminished function of CD8 cytotoxic lymphocytes in killing tumor cells, weakened T-lymphocyte-mediated antitumor responses, and a reduced overall antitumor capacity of the body. A reduction in lymphocyte count in the blood has been identified as an independent prognostic factor for various cancers.^[26]^ Consequently, a high PLR may imply low antitumor immunity, which is associated with an increased cancer risk.

Our study revealed a nonlinear dose-response relationship between the natural logarithm of the PLR (ln (PLR)) and breast cancer (*P* <.05), characterized by an ‘L’-shaped curve. In contrast, the nonlinearity of the natural logarithm of the Systemic Immune-Inflammation Index (ln(SII)) was not significant (*P* >.05). The minimum risk point for ln (PLR) was 4.32; below this threshold, there was a negative correlation, whereas above it, there was a positive correlation. Notably, 80% of the ln (PLR) values are concentrated between 4.32 and 5.214. These findings further underscore the significant association between elevated PLR and an increased risk of breast cancer.

Patients with obesity often experience chronic inflammation. Studies have demonstrated that weight loss can prevent the development of cancer and other obesity-related diseases, and dietary interventions can significantly reduce breast cancer mortality.^[27]^ Additionally, obese patients undergoing bariatric surgery have a decreased risk of breast cancer.^[28]^ Furthermore, medications such as metformin, thiazolidinediones, anti-inflammatory drugs, and other weight-loss agents also lower the risk of breast cancer.^[29,30]^ BMI is a simple calculation based on an individual’s height and weight, with a high BMI indicating a high body fat content. Our study revealed that higher BMI was associated with a greater proportion of individuals with malignant breast tumors. We further conducted subgroup analyses to investigate whether there were differences in the association between ln (SII) and ln (PLR) (natural logarithms of the Systemic Immune-Inflammation Index and PLR, respectively) and breast cancer risk across the different BMI categories. However, the results did not show any significant correlations. Interaction tests only indicated that higher SII and PLR values were more likely to be associated with breast cancer in individuals with a normal BMI; however, there were no significant differences across the overall BMI categories. Similar results were observed in other subgroups, emphasizing the robustness of our findings.

The results of our subgroup analysis revealed a significant interaction between the natural logarithm-transformed PLR (ln-PLR), InSII, and family monthly poverty level (interaction *P* <.05). Previous studies have demonstrated a positive association between high socioeconomic status and breast cancer risk.^[31–33]^ Socioeconomic status (SES) encompasses multiple dimensions such as education level, occupation, and income, with income directly determining the Family Monthly Poverty Level Classification (FMPLC).^[34]^ This study found that the Family Monthly Poverty Level (FMPLC) significantly modifies the relationship between PLR and breast cancer risk (interaction *P* = .031). Baseline data showed that the breast cancer proportion was highest in the low-income group (FMPLC ≤ 1.30) (31.72% vs 26.85% in the noncancer group). However, multivariate regression analysis revealed a more complex pattern: the association between PLR and breast cancer risk was strongest in the middle-income population (FMPLC 1.30–1.85), followed by the high-income group (FMPLC, >1.85). This suggests that socioeconomic factors may operate through different mechanisms. Low-income populations may have low screening rates because of poor medical access, whereas middle-income women often lose eligibility for medical assistance and face delayed cancer screening because of insufficient insurance coverage.^[35–37]^ Additionally, the frequency of p53 mutations was significantly higher in the middle-income group (18%) than in the high-income group (5%; *P* <.05). Abnormal p53 drives inflammatory responses in the tumor microenvironment, which enhances the predictive sensitivity of inflammatory markers such as PLR.^[38]^ Meanwhile, this group lacked the timely intervention capabilities available to the high-income group. Compared to the low-income group, they are more exposed to pro-inflammatory environments, including work stress and delays in diagnosis and treatment.^[39,40]^ This indicates that we still need to further explore the complex associations between individual health behaviors, socioeconomic structure, and inflammatory markers.

## 5. Conclusions

In summary, our research findings conclusively demonstrate a significant correlation between systemic immune-inflammation indices, namely the PLR, and the risk of breast cancer. Specifically, elevated PLR, but not SII, was independently associated with increased breast cancer prevalence. Notably, increased PLR is a crucial factor that significantly increases the risk of breast cancer. Importantly, the relationship between PLR and breast cancer revealed a nonlinear pattern, indicating a complex interplay that does not strictly follow a monotonic trend. Consequently, we underscored the pivotal significance of PLR in predicting the risk of breast cancer. However, to fully comprehend the underlying mechanisms, further investigations aimed at elucidating the causal link between these systemic immune-inflammation biomarkers and breast cancer are indispensable for future research.

## Author contributions

**Conceptualization:** Yanting Li.

**Data curation:** Yanting Li, Xurui Li, Peiling Zhu, Mengsi Zhou.

**Formal analysis:** Yanting Li, Yanpeng Li, Xurui Li, Peiling Zhu.

**Funding acquisition:** Yanting Li, Mengsi Zhou.

**Investigation:** Yanting Li, Yanpeng Li.

**Methodology:** Yanting Li, Yanpeng Li.

**Project administration:** Mengsi Zhou.

**Resources:** Yanting Li, Yanpeng Li, Xurui Li.

**Software:** Yanting Li, Xurui Li.

**Supervision:** Mengsi Zhou.

**Validation:** Yanpeng Li, Peiling Zhu.

**Visualization:** Yanpeng Li, Xurui Li, Peiling Zhu.

**Writing – original draft:** Yanting Li, Mengsi Zhou.

**Writing – review & editing:** Yanting Li, Mengsi Zhou.




