# Supplementary material for: Elevated platelet-to-lymphocyte ratio is associated with increased breast cancer risk: A cross-sectional analysis of the NHANES 2009 to 2020
Source: Medicine (Baltimore). 2026 Jun 19;105(25):e49311. doi: 10.1097/MD.0000000000049311 (PMC13286514; doi:10.1097/MD.0000000000049311)
Supplement: Supplementary file 1 [file medi-105-e49311-s001.docx]

TABLE S1 The relationship between ln-transformed SII and the risk of Breast cancer.

| Characteristic | Crude model ^a^ | | Model 1^b^ | | Model 1^c^ | |
| --- | --- | --- | --- | --- | --- | --- |
|  | OR(95% CI) | P-value | OR(95% CI) | P-value | OR(95% CI) | P-value |
| SII (ln-transformed) | 1.31 (0.98, 1.76) | 0.070 | 1.26 (0.93, 1.72) | 0.133 | 1.24 (0.87, 1.78) | 0.221 |
| In(SII) (Quartile) | | | | | | |
| Q1 | Ref |  | Ref |  | Ref |  |
| Q2 | 1.41 (0.96, 2.07) | 0.076 | 1.44 (0.97, 2.14) | 0.073 | 1.40 (0.81, 2.39) | 0.226 |
| Q3 | 0.93 (0.60, 1.42) | 0.740 | 0.97 (0.63, 149) | 0.903 | 0.95 (0.56, 1.62) | 0.852 |
| Q4 | 1.61 (1.11, 2.35) | 0.014 | 1.56 (1.06, 2.30) | 0.026 | 1.57 (0.92, 2.68) | 0.102 |
| P for trend |  | 0.078 |  | 0.118 |  | 0.224 |

OR, odds ratio; CI, confidence interval; Q, quartile; SII, systemic immune-inflammation index; PLR, platelet-to-lymphocyte ratio.

a The crude model was not adjusted for any covariates.

b Model 1 was adjusted for age and race.

c Model 2 was adjusted for all covariates based on model 1.
