# Supplementary material for: Elevated platelet-to-lymphocyte ratio is associated with increased breast cancer risk: A cross-sectional analysis of the NHANES 2009 to 2020
Source: Medicine (Baltimore). 2026 Jun 19;105(25):e49311. doi: 10.1097/MD.0000000000049311 (PMC13286514; doi:10.1097/MD.0000000000049311)
Supplement: Supplementary file 2 [file medi-105-e49311-s002.docx]

TABLE S2 The relationship between ln-transformed (SII and PLR) with Breast cancer risk in different subgroups.

| Characteristic | SII (ln-transformed) | | PLR (ln-transformed) | |
| --- | --- | --- | --- | --- |
|  | OR (95%CI) | P-int | OR (95%CI) | P-int |
| Age |  | 0.973 |  | 0.295 |
| 20-39 years | 1.36 (0.85, 2.19) |  | 1.18 (0.60, 2.33) |  |
| 40-59 years | 1.43 (0.94, 2.19) |  | 1.71 (0.91, 3.21) |  |
| ≥60 years | 1.33 (0.85, 2.08) |  | 2.51 (1.30, 4.82) |  |
| Race |  | 0.683 |  | 0.950 |
| Mexican American | 1.55 (0.76, 3.14) |  | 2.10 (0.76, 5.80) |  |
| Other Hispanic | 1.93 (0.86, 4.30) |  | 1.18 (0.36, 3.87) |  |
| Non-Hispanic White | 1.18 (0.81, 1.73) |  | 1.65 (0.95, 2.87) |  |
| Non-Hispanic Black | 1.30 (0.75, 2.25) |  | 2.02 (0.92, 4.43) |  |
| Other Races | 2.12 (0.84, 5.36) |  | 1.75 (0.42, 7.31) |  |
| BMI |  | 0.078 |  | 0.146 |
| <25 Kg/m^2^ | 1.13 (0.67, 1.88) |  | 1.05 (0.49, 2.23) |  |
| 25-30 Kg/m^2^ | 2.29 (1.37, 3.83) |  | 3.06 (1.44, 6.53) |  |
| ≥30 Kg/m^2^ | 1.18 (0.81, 1.70) |  | 1.75 (1.02, 3.00) |  |
| Education level |  | 0.862 |  | 0.721 |
| Less than high school | 1.52 (0.61, 3.75) |  | 1.77 (0.48, 6.46) |  |
| High school or equivalent | 1.48 (0.98, 2.24) |  | 2.09 (1.15, 3.81) |  |
| Some college or more | 1.29 (0.90, 1.84) |  | 1.51 (0.89, 2.54) |  |
| WC (Quartile) |  | 0.574 |  | 0.877 |
| Q1 | 1.27 (0.68, 2.37) |  | 1.49 (0.59, 3.74) |  |
| Q2 | 1.44 (0.83, 2.50) |  | 1.39 (0.63, 3.09) |  |
| Q3 | 1.78 (1.09, 2.92) |  | 2.06 (1.02, 4.19) |  |
| Q4 | 1.11 (0.71, 1.74) |  | 1.88 (0.98, 3.61) |  |
| FMPLC |  | 0.050 |  | 0.031 |
| ≤1.30 | 1.01 (0.68, 1.51) |  | 0.99 (0.55, 1.78) |  |
| 1.30-1.85 | 2.88 (1.29, 6.41) |  | 3.57 (1.18, 10.75) |  |
| ＞1.85 | 1.57 (1.08, 2.28) |  | 2.53 (1.45, 4.39) |  |
| Childbearing history |  | 0.558 |  | 0.597 |
| Yes | 1.37 (1.05, 1.79) |  | 1.73 (1.17, 2.55) |  |
| No | 2.07 (0.53, 8.10) |  | 2.82 (0.48, 16.67) |  |
| Use of estrogen medications |  | 0.489 |  | 0.827 |
| Yes | 1.15 (0.62, 2.10) |  | 1.93 (0.78, 4.77) |  |
| No | 1.45 (1.09, 1.93) |  | 1.72 (1.14, 2.61) |  |
| Hypertension |  | 0.683 |  | 0.662 |
| Yes | 1.30 (0.89, 1.90) |  | 1.90 (1.09, 3.31) |  |
| No | 1.45 (1.02, 2.05) |  | 1.61 (0.96, 2.69) |  |
| Hyperlipidemia |  | 0.967 |  | 0.108 |
| Yes | 1.37 (0.97, 1.95) |  | 1.30 (0.78, 2.18) |  |
| No | 1.39 (0.95, 2.03) |  | 2.42 (1.39, 4.19) |  |
| Diabetes |  | 0.408 |  | 0.419 |
| Yes | 1.11 (0.63, 1.96) |  | 1.28 (0.56, 2.94) |  |
| No | 1.45 (1.08, 1.94) |  | 1.88 (1.23, 2.87) |  |
| PHQ-9 score |  | 0.684 |  | 0.159 |
| 0-4 | 1.49 (1.08, 2.06) |  | 2.35 (1.46, 3.78) |  |
| 5-9 | 1.12 (0.62, 2.00) |  | 0.98 (0.42, 2.29) |  |
| ≥10 | 1.51 (0.77, 2.96) |  | 1.28 (0.50, 3.31) |  |

OR, odds ratio; CI, confidence interval; P-int, P for interaction; SII, systemic immune-inflammation index; PLR, platelet-to-lymphocyte ratio; WC: Waist circumference; FMPLC: Family monthly poverty level category; Q, quartile.
